# Best videos of the year in 2017

**DOI:** 10.1590/S1677-5538.IBJU.2018.0002

**Published:** 2018

This year has been another memorable year for the *International Brazilian Journal of Urology* with quality international submissions to the video section from across the World. It highlights the commitment by the urology community in continually striving to improve the outcomes of surgical patients. We should never accept that good is good enough particularly with the plethora of surgical tools at our disposal. In this regard, it gives me a distinct pleasure to introduce the three best videos for 2017. These selections were made based on their novelty and potential to favorably impact the surgical care of urological patients. Making these selections is always challenging, being that all accepted videos are already of an incredibly high quality; hence, I would like to congratulate all authors for accepted videos over the past year and look forward to receiving your future submissions in the months to years to come. The selection of best videos of the year are as follows:

The third prize goes to the video entitled “Laparoscopic partial nephrectomy for multiple (four) tumors” by Lessandro Curcio et al. from the Hospital Federal Ipanema in Brazil, published in the May-June issue (43(3): 567) (1) of our Journal. The authors nicely depict in this video a two-stage approach to managing a patient with multifocal renal tumors using a minimally invasive laparoscopic approach in which the larger tumors were approached in an initial setting using a conventional clamp technique, followed by which the two smaller ones were approach in a non-clamped manner. This video nicely depicts that each case must be approached in a personalized fashion with the individual tumor characteristics, multifocality, and location aiding in the selection of the most suitable surgical strategy, including appropriateness of minimally invasive and clamp versus non-clamp vascular hilar control techniques.

The second prize is awarded to the video entitled “Feasibility of robot-assisted segmental ureterectomy and ureteroureterostomy in a patient with high medical comorbidity” by Raheem Ali Abdel et al. from centers of excellence in Egypt and South Korea (published in the Jul-Aug issue (43(4): 779-80) (2). This surgical resection was completed with meticulous oncological technique using a robotic assisted minimally invasive approach. The surgery was completed with minimal blood loss and with a reported total surgical time of only 100 minutes. This video nicely depicts that such distal ureteral tumors can be resected with negative surgical margins and utilizing an appropriate ureteral reconstruction technique. The accepted abstract for this video was accompanied by a very nice editorial by Dr. Alejandro Rodriguez who highlights important considerations in such cases.

The first and top prize is awarded to Peter Caputo et al. from the Cleveland Clinic in Ohio in their video entitled “Robotic assisted laparoscopic augmentation ileocystoplasty” which was published in the Sept-Oct issue of our Journal (43(5): 994) (1). This video is innovative in its approach, with a truly beautifully video depiction with excellent accompanying narration. It truly encompasses all of the qualities and characteristics we seek in truly benchmark video submissions with originality, superior quality of surgical video clips and narration, along with high educational value for our readership.

Finally, I would like to thank each one of you for your continual support of the video section of the *International Brazilian Journal of Urology,* ultimately insuring its growing inter-national success and regard among the superior peer reviewed urological journals. On behalf of our entire editorial team, we hope each of you and your family’s had happy holidays and the very best wishes for the upcoming year.
